# 

**Published:** 1904-10

**Authors:** 


					﻿Sixtieth Year.
OCTOBER. 1904.
BUFFALO
MEDICAL JOURNAtf
VOL. LX.
Whole Number,
DCLXXXX\
New Series.
VOL. XLIV
No. 3
Established 1845 by AUSTIN FLINT, M. D.
aOrUCc : editor:
uyjjLLIAM WARREN POTTER, M. D.
ASSISTANT EDITORS:
WILLIAM C. IWAUSS, M. D.
NELSON W. WILSON, M. D.
’‘ASSOCIATE EDITORS:
JAMES WRIGHT PUTNAM, M. D.
aHJ^SAflMlEjITER, M. D.
^HARVEY R. GAYLORD, M. D.
ERNEST WENDE, M. D.
JOHN A. MILLER, Ph. D.
MAUD J. FRYE, M. D.
For terms and other information relating to the Journal, see page vi.
				

